# Exploring the relationship between North Star Ambulatory Assessment and Health Utilities Index scores in Duchenne muscular dystrophy

**DOI:** 10.1186/s12955-023-02160-8

**Published:** 2023-07-19

**Authors:** Ivana Audhya, Basia Rogula, Shelagh M. Szabo, David Feeny, Talshyn Bolatova, Katherine Gooch

**Affiliations:** 1 Sarepta Therapeutics, Inc., Cambridge, MA USA; 2 Broadstreet HEOR, 201 – 343 Railway St, Vancouver, BC V6A 1A6 Canada; 3 McMaster University and Health Utilities Inc, Hamilton, ON Canada

**Keywords:** Duchenne muscular dystrophy, Utility, NSAA, HUI

## Abstract

**Background:**

The North Star Ambulatory Assessment (NSAA) documents motor performance in ambulatory individuals with Duchenne muscular dystrophy (DMD). Health Utilities Index (HUI) scores, reflecting preferences for health-related quality-of-life (HRQoL) implications of health states, are commonly estimated within trials. This study sought to characterize the relationship between the NSAA score and utility in DMD.

**Methods:**

Family members serving as proxy respondents for placebo-treated ambulatory individuals with DMD (NCT01254019; BioMarin Pharmaceuticals Inc) completed the HUI and the NSAA (score range, 0–34). Mean change over time on these measures was estimated, and the correlation between changes in NSAA score and a) HUI utility; b) HUI3 ambulation and HUI2 mobility attribute scores, over 48 weeks was calculated.

**Results:**

Baseline mean (range) age was 8.0 years (5–16; *n* = 61) and mean (standard deviation [SD]) scores were 0.87 (0.13; HUI2), 0.82 (0.19; HUI3), and 21.0 (8.1; NSAA). Mean (SD) change over 48 weeks was –0.05 (0.14; HUI2), –0.06 (0.19; HUI3), and –2.9 (4.7; NSAA). Weak positive correlations were observed between baseline NSAA score and HUI utility (HUI2: *r* = 0.29; HUI3: *r* = 0.17) and for change over 48 weeks (HUI2: *r* = 0.16; HUI3: *r* = 0.15). Stronger correlations were observed between change in NSAA score and the HUI3 ambulation (*r* = 0.41) and HUI2 mobility (*r* = 0.41) attributes.

**Conclusions:**

Among ambulatory individuals with DMD, NSAA score is weakly correlated with HUI utility, suggesting that motor performance alone does not fully explain HRQoL. Stronger relationships were observed between HUI ambulation and mobility attributes, and NSAA. Although unidimensional measures like the NSAA are informative for documenting disease-specific health impacts, they may not correlate well with measures of overall health status; requiring use in conjunction with other patient-reported and preference-based outcomes.

**Supplementary Information:**

The online version contains supplementary material available at 10.1186/s12955-023-02160-8.

## Introduction

Duchenne muscular dystrophy (DMD) presents in early childhood with muscle weakness, gait abnormalities, impaired posture, and delayed motor function [1–5]. Progressive degeneration of muscle cells and development of muscular weakness leads to loss of ambulation in the pre-teenage and early teenage years. Over time, patients further succumb to loss of upper limb function, giving rise to functional dependence, respiratory insufficiency, progressive decline in cardiac function, and premature mortality [6]. These symptoms of DMD impact patient comfort, activities of daily living, and health-related quality-of-life (HRQoL) [7]. DMD is therefore a complex chronic condition that significantly impacts both patients and their families/caregivers due to the severity of symptoms, its progressive nature, the impact of caregiving tasks, and the associated loss of functional independence [8].

Given the impact loss of motor function has on functional independence and HRQoL for those with DMD, maintaining the ability to ambulate is an important treatment goal; therefore, assessing motor and ambulatory function are important endpoints [9]. The North Star Ambulatory Assessment (NSAA) is a validated and reliable instrument that measures changes in motor performance (such as ability to rise from the floor (RFF), move from sitting to standing, jump, run, walk, and ascend/descend steps) in ambulant individuals with DMD [10–12]. The NSAA may be used in combination with timed function tests (TFTs) to further assess different aspects of changes in motor performance. Some studies have reported correlations between NSAA scores and TFTs [13–15]. While one study has shown that NSAA score and other measures of physical function (e.g., 6-minute walk test [6MWT] distance, as well as other TFTs) are correlated with selected measures of HRQoL at baseline, this relationship did not hold when considering 12-month changes [16]. Moreover, other data to directly describe the relationship between performance on functional motor assessments in DMD and HRQoL scores are presently unavailable.

*Utility values* reflect individual preferences for the HRQoL impact of a given health state or health outcome. These are represented as values anchored from 0 (dead) to 1 (perfect health) [17] and are used in economic evaluations to quality-adjust life years for patients on a particular treatment [18]. Economic evaluations are conducted to understand the potential ‘value for money’ of interventions for a given health condition [19]. Several standardized and comprehensive generic preference-based instruments have been developed for valuating HRQoL, including the Health Utilities Index (HUI), Short-Form 6D, and the EuroQoL Group’s EQ-5D survey. These instruments include a generic HRQoL questionnaire and an accompanying formula or set of weights elicited from a sample of the general population for converting responses into utility values.

Existing data on health state utility in DMD [20, 21] are largely derived from the EQ-5D and the HUI, and a systematic review identified five studies that published health state utility from the perspectives of patients with DMD and their caregivers [20]. Patient and caregiver (or patient-proxy) utility estimates deteriorate significantly between early ambulatory and late non-ambulatory health states [7, 22]. A recent study evaluated changes in health state utility over time and predictors of utility in DMD using the HUI [23]. However, to date, little has been reported on the association between clinical and functional outcome measures, such as the NSAA, and patient HRQoL (measured by generic utility measures, like the HUI).

For DMD, higher utility values would be expected in patients with greater functional abilities and lower utilities in patients with lesser functional abilities. While data are not presently available for DMD, this relationship has been observed between *ambulatory* function tests (like the 6MWT) and multidimensional measures of utility in other therapeutic areas (including muscular dystrophy in adults, cystic fibrosis, heart failure or other pulmonary diseases for example) [24–27]. Whether a similar relationship exists between measures of *motor* performance (such as the NSAA) and utility has not previously been investigated in DMD. The objectives of this study were therefore to describe the relationship between motor performance (measured by NSAA score) and health state utility scores (measured by HUI) over time, among a sample of ambulatory individuals with DMD.

## Methods

Data from placebo-treated ambulant males with DMD aged ≥ 5 years old with exon 51 skip amenable mutations recruited under NCT01254019 (provided by BioMarin Pharmaceutical Inc.) were included. These data were collected as part of a placebo-controlled double-blind randomized trial to assess the safety and efficacy of a novel therapy (drisapersen) for DMD; the clinical protocol inclusion criteria required patients be able to complete the 6MWT of > 75 m at each pre-drug visit. The follow-up period was 48 weeks from baseline (randomization) [28].

The study protocol, informed consent, and other study documents were reviewed and approved by national, regional, or investigational center ethics committee or institutional review boards, as appropriate. The NCT01254019 study was performed in accordance with the International Conference on Harmonisation Good Clinical Practice guidelines, the Declaration of Helsinki (2008), and applicable country-specific requirements. Written informed consents from parents/caregivers and assent (from appropriately aged individuals) were obtained for all study participants prior to any study procedure.

### Outcome measures

To measure utility, family members responding on behalf of patients completed the 15-item HUI questionnaire at screening (proxy reporting), baseline, 24 weeks, and 48 weeks (or early withdrawal). HUI responses were used to derive utility (on a scale of 0 [dead] to 1 [full health]) according to two complementary health-status classification systems: the HUI mark 3 (HUI3) and the HUI mark 2 (HUI2) [29]. The HUI3 system considers eight attributes: vision, hearing, speech, ambulation, dexterity, emotion, cognition, and pain. The HUI2 system considers seven attributes: sensation (vision, hearing, and speech), mobility, emotion, cognition, self-care, pain, and (optionally) fertility. Descriptive levels of impairment range from 1 (no impairment) to 5 or 6 (severe impairment) for the eight HUI3 attributes and from 1 (no impairment) to 4 or 5 (severe impairment) for the six relevant HUI2 attributes (as fertility was excluded). Therefore, for both HUI3 and HUI2 attributes, increasing attribute levels indicate worsening function. HUI3 and HUI2 attribute levels are transformed using the developers’ algorithm to generate an overall HUI3 and HUI2 utility score [30]. “Single-attribute” scores of morbidity provide insight into health deficit measured by each attribute and range from 0 to 1. The clinically important change in overall HUI utility scores is 0.03 [29]. An outline of the HUI scoring algorithm is provided in Supplementary Fig. 1.

To measure motor performance, the NSAA was conducted by trained assessors at baseline and every 12 weeks for 48 weeks. The NSAA is a 17-item functional assessment scale designed for evaluating ambulant boys with DMD, where performance on tests related to motor performance (e.g., box climbing, lifting head, running) is assessed [11]. Scores range from 0 to 34, with higher scores indicating better motor performance. Each item is scored on a 3-point scale (0 = unable to achieve independently; 1 = modified method but achieves goal independent of physical assistance from another; and 2 = normal – no obvious modification of activity) [11]. Total scores are calculated by summing the scores for the individual items. The validity, feasibility, and reliability of the NSAA have previously been demonstrated [10, 12, 14, 31]. It has been suggested that the clinically important difference in NSAA scores over time is approximately 2.3 points [32, 33].

Additional measures of ambulatory function conducted at baseline and every 12 weeks for 48 weeks included the 6MWT, RFF, and timed 10-meter walk/run (10MWR) tests. The 6MWT measures the total distance (in meters) walked by an individual on a flat hard surface within 6 min [34]. The RFF test measures the time taken to rise from a standardized supine position to upright standing [11]. The timed 10MWR test measures the time spent walking/running 10 m as quickly as possible while ensuring that the participant is safe [11]. For both the 10MWR and RFF tests, a grading code from 1 to 6 can be assigned based on performance on the tests. These grading codes have the advantage of being able to assign values to patients for whom timed test values cannot be recorded due to physical inability. Definitions and explanations of scoring by measure are outlined in the Supplementary Table.

### Analysis

Demographics at baseline were summarized. HUI3 and HUI2 attribute levels were calculated for each individual at baseline, 24 weeks, and 48 weeks (where this was possible, given availability of the required responses from the HUI questionnaire at each time point). An individual’s HUI responses could be unavailable due to incomplete, incorrect, or uninformative responses, or a missing assessment; no imputation was performed for visits with missing data. HUI3 and HUI2 utility values were estimated for each individual at these same visits following the developers’ algorithm and scoring instruction [30]. To summarize HUI utility at baseline and over time, mean (standard deviation [SD]) utility values were calculated at baseline, 24 weeks, and 48 weeks [23]. Similarly, mean (SD) NSAA score values were calculated at baseline and every 12 weeks, to 48 weeks. In an exploratory analysis, these mean (SD) values of HUI utility and NSAA score by visit were stratified by age (< 8 years, ≥ 8 years). The mean HUI score, NSAA total score, 6MWT distance, 10MWR grade, and RFF grade at baseline were summarized. Mean *changes* in NSAA score, 6MWT, and HUI score from baseline to 48 weeks were also described.

To explore the relationship between the NSAA and other functional measures, correlations between NSAA score and the 6MWT, RFF grade, and 10MWR grade at baseline were explored. The correlation between 6MWT and HUI2 and HUI3 utility at baseline, and change in scores over time, was also calculated.

Scatterplots with best-fitting lines were used to explore the relationship between NSAA score and HUI utility. Correlations were estimated between NSAA score and HUI score at baseline, as well as correlations between changes in NSAA and HUI scores over 48 weeks. The characteristics of individuals that contribute to variability in the estimate of correlation between NSAA and HUI scores was visually assessed. The correlation between change in NSAA scores and change in HUI3 and HUI2 attribute scores was also explored. A linear regression analysis was run to estimate the change in HUI score associated with a one-unit change in NSAA score. The percent of variation around mean changes in utility explained by changes in NSAA score (from baseline to week 48) was estimated using the R^2^ measure from this linear regression analyses.

Correlations between ordinal measures (NSAA with RFF or 10MWR), or utility and NSAA, were calculated as Spearman’s rank correlations. Pearson’s correlation was used to summarize correlations between utility and 6MWT. All analyses were conducted in R.

## Results

### Characteristics at baseline

Sixty-one ambulant males with DMD were included in this study. At baseline, mean age was 8.0 years (range, 5 − 16). The mean (SD) utility score was 0.82 (0.19) for the HUI3 and 0.87 (0.13) for the HUI2. The mean (SD) NSAA total score was 21.0 (8.1), mean (SD) grade for the 10MWR test was 4.8 (1.0), and mean (SD) grade for the RFF test was 3.3 (1.4) (Table 1). Mean (SD) distance walked in the 6MWT was 348 (92) meters.

**Table 1:** Baseline characteristics

Characteristics	DMD sample (*n* = 61)
**Age, years**	
Mean (SD)	8.0 (2.4)
Median (IQR)	8 (6, 9)
Min, max	5, 16
**By group, *n (%)***^**a**^	
5 − 7 years	29 (47.5)
8 − 11 years	29 (47.5)
12 − 16 years	3 (4.9)
**NSAA total score**	
Mean (SD)	21.0 (8.1)
Median (IQR)	23 (15, 27)
Min, max	4, 33
**6MWT (m)**	
Mean (SD)	348 (92)
Median (IQR)	354 (311, 400)
Min, max	108, 566
**10MWR grade**^b^	
Mean (SD)	4.8 (1.0)
**RFF grade**^b^	
Mean (SD)	3.3 (1.4)

*Abbreviations*: *6MWT* 6-min walk test, *10MWR* 10-m walk/run, *DMD* Duchenne muscular dystrophy, *IQR* Interquartile range, *m* meters, *max* maximum, *min* minimum, *NSAA* North Star Ambulatory Assessment, *RFF* Rise from floor, *SD* Standard deviation

^a^While age categories 8 − 11 years and 12 − 16 years were initially investigated, the three patients in the 12 − 16 years category were considered along with the 8 − 11 year old boys, due to small sample size

^b^*n* = 55

### Correlation between measures at baseline

**Fig. 1:**
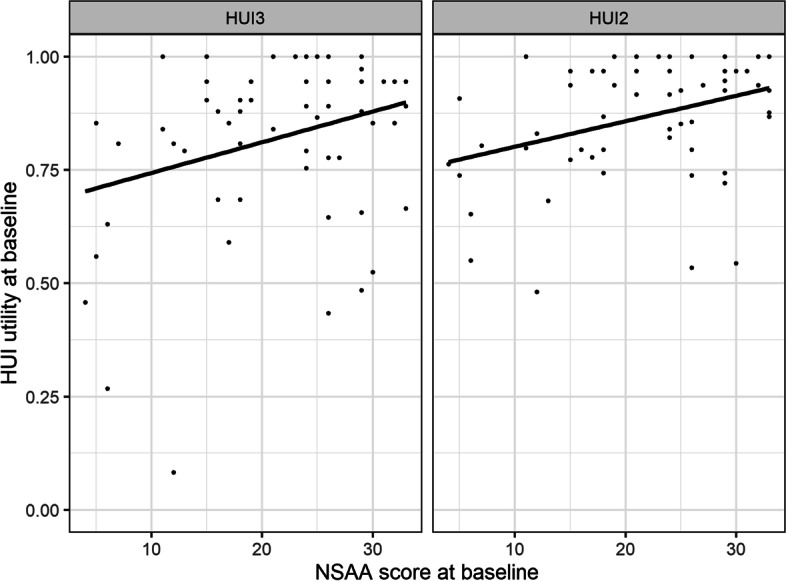
Scatterplots of HUI utility vs. NSAA score at baseline, with best-fitting lines. Abbreviations: HUI, Health Utility Index; HUI2, Health Utility Index mark 2; HUI3, Health Utility Index mark 3; NSAA, North Star Ambulatory Assessment

At baseline, the correlation was strong between NSAA scores and 10MWR grades (*r* = 0.80), between NSAA scores and RFF grades (*r* = 0.79), and between NSAA and 6MWT scores (*r* = 0.69; Supplementary Fig. 2). Weak positive correlations were observed between NSAA scores and HUI scores at baseline (HUI3: *r* = 0.17; HUI2: *r* = 0.29; Fig. 1). Moderate positive correlations were observed between 6MWT and HUI scores at baseline (HUI3: *r* = 0.39; HUI2: *r* = 0.43; Supplementary Fig. 2).

### Change in NSAA, HUI and 6MWT from baseline to 48 weeks

**Fig. 2:**
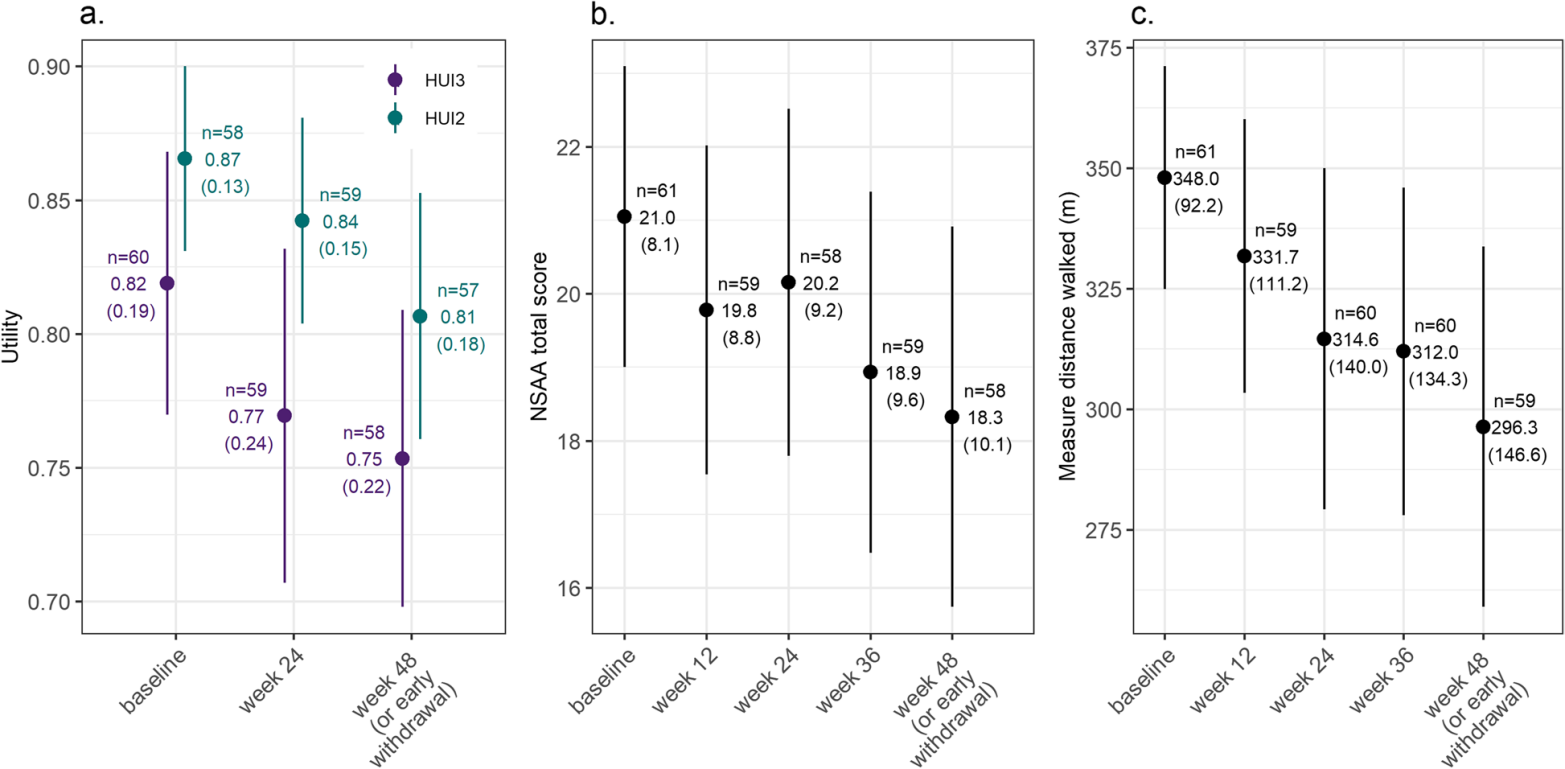
Mean (SD) **A** HUI utility **B** NSAA and **C** 6MWT score by visit. Note: Whiskers represent 95% confidence intervals. Abbreviations: 6MWT: 6-min walk test; HUI, Health Utility Index; HUI2, Health Utility Index mark 2; HUI3, Health Utility Index mark 3; NSAA, North Star Ambulatory Assessment; SD, standard deviation

HUI scores decreased over time, with a mean (SD) utility score at week 48 (or early withdrawal) of 0.75 (0.22) for the HUI3 and 0.81 (0.18) for the HUI2 (Fig. 2). This was a decrease of 0.06 (0.19) and 0.05 (0.14), respectively, among those with non-missing values at both baseline and week 48. NSAA scores, which were variable between individuals, also decreased over time to a mean NSAA score of 18.3 (10.1) at week 48, reflecting a decrease of 2.9 (4.7) from baseline among those with non-missing values at both baseline and week 48 (Fig. 2). Changes were greater on average among older (≥ 8 years) vs. younger (< 8 years; Supplementary Fig. 3) individuals. 6MWT scores also decreased over time, with a mean (SD) distance at week 48 (or early withdrawal) of 296.36 (146.59) meters. Changes were also greater on average among older (≥ 8 years; -83.7 (96.4) meters) vs. younger (< 8 years; -23.8 (59.1) individuals.

### Correlation between NSAA and HUI, and 6MWT and HUI

**Fig. 3:**
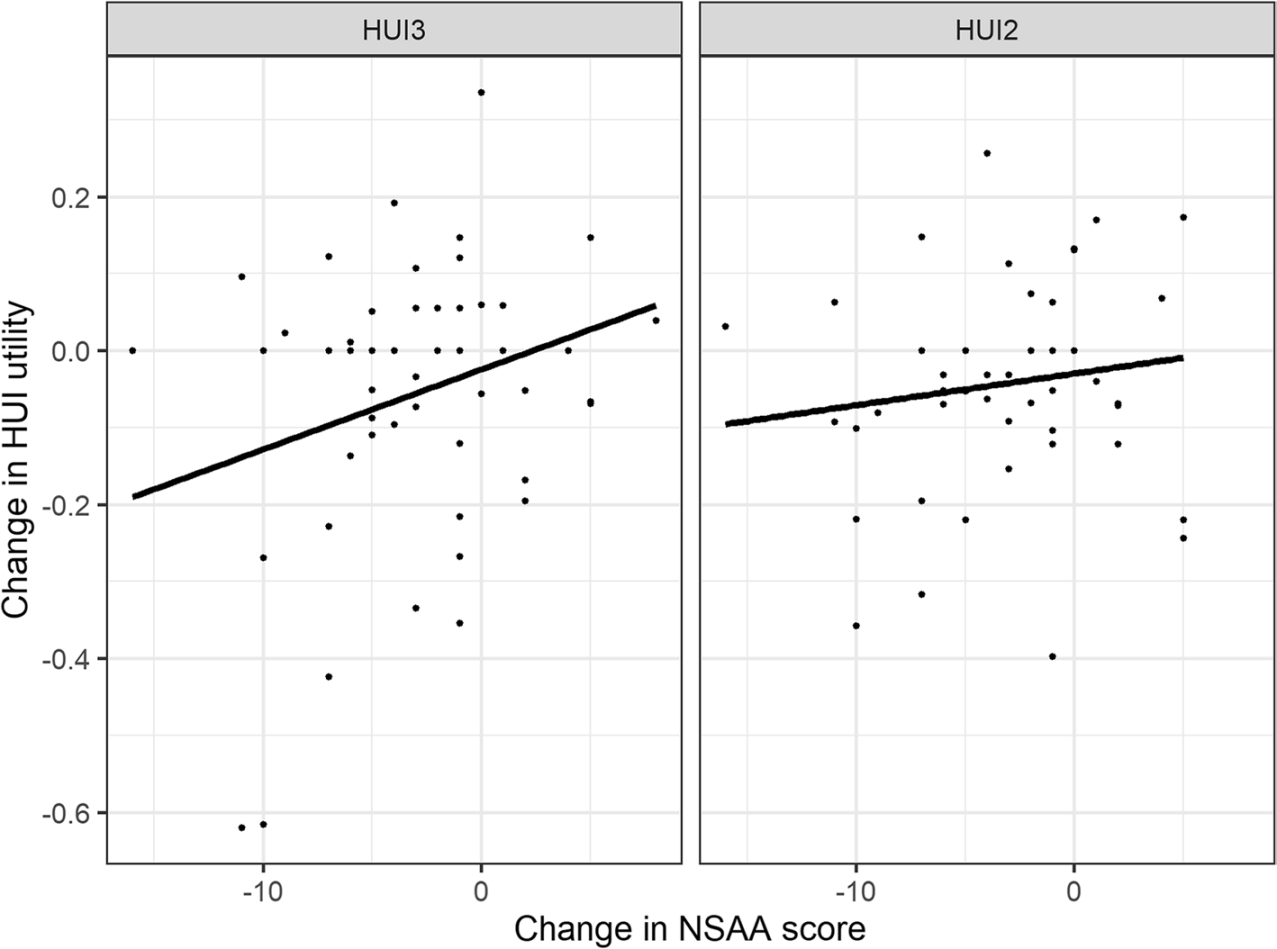
Change (week 48—baseline) in HUI utility vs. NSAA score, with best-fitting lines. Abbreviations: HUI, Health Utility Index; HUI2, Health Utility Index mark 2; HUI3, Health Utility Index mark 3; NSAA, North Star Ambulatory Assessment

Weak positive correlations were observed between the change in NSAA scores and HUI scores over 48 weeks (HUI3: *r* = 0.15; HUI2: *r* = 0.16; Fig. 3). A one-unit decline in NSAA score was associated with a 0.010 decline in HUI3 utility and a 0.004 decline in HUI2 utility. These are represented by the slopes of the best-fitting lines in Fig. 3. The analyses of the percent of variation around mean change in utility explained by change in NSAA score demonstrated that change in NSAA score explained 7% of variability in change in HUI3 scores and 2% of variability in change in HUI2 scores. Weak to moderate correlations were also noted between change in HUI2 and HUI3 utility and change in 6MWT over 48 weeks (HUI2: *r* = 0.35, HUI3: *r* = 0.41).

### Correlation between NSAA and particular HUI attributes

**Fig. 4:**
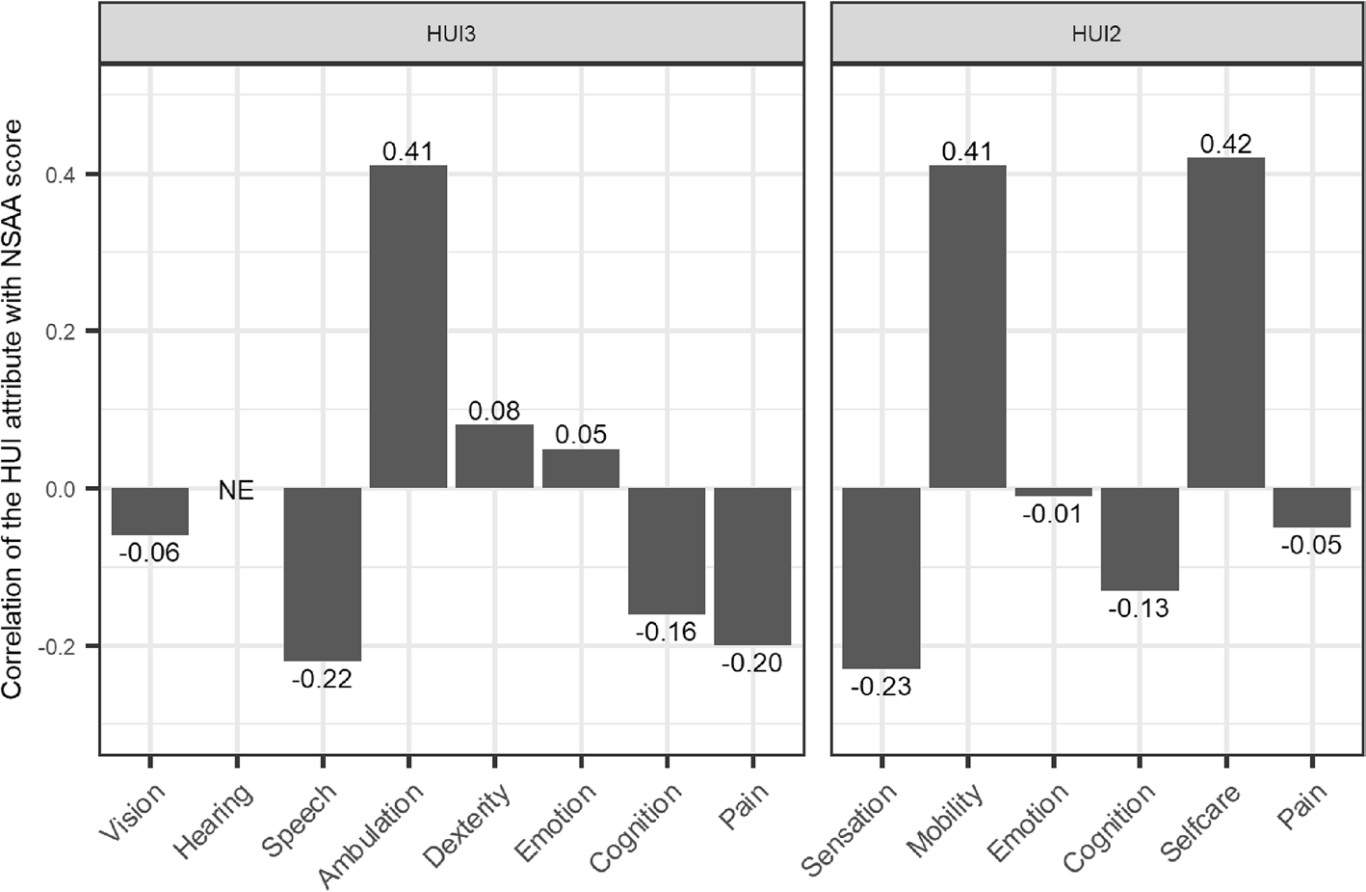
Spearman correlation of NSAA scores with individual HUI attribute scores, across all visits. Abbreviations: HUI, Health Utility Index; HUI2, Health Utility Index mark 2; HUI3, Health Utility Index mark 3; NE, not estimable; NSAA, North Star Ambulatory Assessment

Moderate positive correlations were observed between NSAA scores and the HUI3 ambulation (*r* = 0.41), HUI2 mobility (*r* = 0.41), and HUI2 self-care (*r* = 0.42) scores across all visits; NSAA scores were weakly negatively correlated with HUI3 pain (*r* =  − 0.20; Fig. 4). Visual inspection of scatterplots revealed potential sources of variability that would impact the correlation between NSAA scores and HUI utility values. These sources included individuals with high HUI scores and low levels of function at baseline (Fig. 1), as well as those with large changes in utility and little change in NSAA score (Fig. 3). Specifically, three individuals with relatively stable NSAA scores (change ≤ 1) had substantial declines in HUI3 utility of at least 0.2 due to worsening in attributes, including emotion (1 patient worsened 2 levels), cognition (2 patients worsened 2 levels), and dexterity (1 patient worsened 2 levels). One individual with a small decline in NSAA score of 1 had a large decline in HUI2 utility of 0.40 due to emotion worsening 3 levels and pain worsening 1 level.

## Discussion

Chronic progressive neuromuscular diseases such as DMD frequently cause reduced ambulatory function and mobility and are known to give rise to diminished HRQoL, and resultant utility values, over time [6–8, 20]. However, few studies have published utility values for DMD health states, and those that exist have only characterized health states by age, ambulatory status, and need for ventilation [20]. There are no utility values for health states that are defined by intermediate clinical endpoints such as the NSAA, which measures gross motor performance among ambulatory individuals with DMD.

In the current study, a strong correlation was observed between NSAA scores and TFTs (10MWR and RFF grades, in addition to 6MWT distance), supporting the value of the NSAA as a measure of motor performance. Other studies have found similar associations between NSAA score and TFTs [13–15]. In 2010, Mazzone et al. [14] assessed functional performance in a cohort of 112 ambulant males with DMD and reported NSAA score had a moderate to good correlation with the 6MWT (*ρ* = 0.589; *p* < 0.01) and the RFF (*ρ* =  − 0.711; *p* < 0.01) but less with the 10MWR (*ρ* =  − 0.505; *p* < 0.01). To better understand the natural history of NSAA scores in those with DMD, Muntoni et al. [13] performed a latent class analysis to identify sets of trajectories based on ambulatory function as measured by NSAA score. Grouping individuals by NSAA-based latent classes, the study found that patterns of decline on the 10MWR and RFF tests were concordant with that of NSAA total score.

Results of the current study showed moderate positive correlations between NSAA score and particular HUI attributes, including HUI3 ambulation, HUI2 mobility, and HUI2 self-care attributes. This finding speaks to criterion validity, as the degree and direction of correlation was expected given that the NSAA and the specified HUI attributes measure similar underlying target concepts.

However, the correlation between baseline NSAA and HUI scores, and in changes in these scores over time, was weak. There are several potential hypotheses to explain this. First, the relationship between ambulatory function or mobility, motor performance, overall health status, and HRQoL is not straightforward. For example, individuals may report improved HRQoL after the occurrence of functional loss accompanying major disease milestones in DMD (such as loss of ambulation) due to adaptation where patients accommodate to their changed functional status [23, 35]. It is notable that the relationship between ambulatory function (measured by the 6MWT) and utility was stronger than that observed between motor performance test ( the NSAA) and utility; suggesting that ambulation may be more important for overall HRQoL than performance on specific motor-related tasks. Second, the underlying construct that is being assessed related to ambulation or mobility, differs between the NSAA and HUI instruments. Ambulation within the HUI3 is based on patients’ ability to navigate their neighborhood. Mobility is conceptualized within the HUI2 as a combination of ambulatory function and dexterity, and because of the importance of upper limb function for dexterity [35], it therefore may have more relevance to those with DMD. In contrast, the NSAA specifically assesses motor performance, not solely ability to ambulate. Third, although the reliability of the HUI has been demonstrated, scores may ultimately be influenced by a variety of other factors, such as the source of report. It is therefore possible that utility as reported by the patients themselves might differ from reports provided by proxy respondents. Fourth, the age-specific analyses of change in HUI and NSAA scores over time highlighted that younger individuals in particular did not experience great changes in either ambulatory function or utility over time; a large proportion of the study sample was in that age category. That little change is observed in ambulatory function among those of younger ages is consistent with what one would expect from the known natural history of DMD [6]. However, a greater change in ambulatory function and utility among individuals in this subgroup would afford a greater ability to detect meaningful correlations. Finally, although large for a study in rare diseases, the small sample size in this study limited the ability to further explore the relationship between functional measures and utility and may have contributed to the weak correlation observed.

The study findings support that changes in NSAA score alone are not necessarily a sole predictor of change in HUI utility and vice versa. The HUI documents health status using a multi-attribute health status classification system, including aspects of ambulatory, physical, cognitive, and sensory function; social and emotional health; and attributes such as ability to complete self-care activities and presence of pain [29]. A previous analysis using the same sample with DMD found that the attributes of pain and emotion along with ambulation/mobility and self-care were the best predictors of changes in utility, as they explained the largest proportion of variability in changes in utility [23]. These data support that additional factors feed into determining HUI utility in DMD, with utility declines being multifactorial and reflecting the impact of numerous aspects of patient well-being.

The HUI by design is a generic, multidimensional health status assessment instrument, and such instruments are not always sufficient for measuring disease-specific impacts in a given population [21]. The DMD-QoL is a novel condition-specific HRQoL measure that was developed through Project Hercules, based on direct patient and caregiver feedback on what is important to those with DMD and their families. Project Hercules envisioned that the DMD-QOL would be used to measure utility values for DMD-specific health states [21, 36]. Given this instrument was developed to measure the specific impacts experienced by those with DMD, it would be interesting to explore its relationship with the NSAA. However, to the best of our knowledge, at the time of this research such an assessment has not yet been presented. Preliminary evidence from the DMD-QOL is suggesting that there is limited difference in utility between many functional health states in DMD{Bever, 2023 #43} – consistent with aspects of the patient testimonies that contributed to the instrument development process – but may result in less sensitivity to change against the NSAA (and as a corollary, have less correlation with the NSAA). The relationship between DMD-QOL and NSAA scores will be an interesting area to investigate with emerging data from the DMD-QOL.

Potential limitations of this study include the use of a relatively small sample size, which limits the statistical power of the analysis. The generalizability of the study may be limited by the fact that the demographic characteristics (e.g., patients were exon 51/53 skip-amenable) were not representative of the wider population of those with DMD; although those who are exon 51/53 skip amenable represent one of the larger mutation subgroups with DMD, their phenotype may be more severe than some others in terms of time to loss of ambulation [37]. Nonetheless, even among ambulatory individuals with DMD, timing of declines in function would vary from patient to patient and observed trajectories need to be interpreted in that context. As previously mentioned, utilities were based on caregivers serving as proxy respondents, which may not as accurately reflect the patient’s preference as would those that were self-reported. Finally, as these were clinical trial participants, even though they were allocated to the placebo group, there is the potential that optimism about potentially receiving an efficacious novel treatment may have impacted outcome assessment.

The results of these analyses highlight that DMD is a multifaceted disease and although functional impairment occurs early in the disease progression, motor performance alone does not fully explain changes in HRQoL in ambulatory individuals with DMD. While NSAA score is correlated with the ambulatory- and mobility-related components of the HUI, it is not strongly correlated with overall HUI utility, suggesting that additional important drivers of utility exist. It is also important to acknowledge that although some domains of the HUI have an appreciable association with the NSAA, overall the two instruments measure different, yet meaningful concepts. It is important to recognize the role of HRQoL and utility, as well as function, in assessing the patient experience in ambulatory children with DMD.

## Supplementary Information

**Additional file 1:** **Supplementary Table.** Measure scoring. **Supplementary Fig. 1.** HUI scoring. *HUI2 level codes for Sensation, Mobility, and Cognition attributes can be derived directly from HUI3 attribute level codes. Abbreviations: HUI, Health Utility Index; HUI2, Health Utility Index mark 2; HUI3, Health Utility Index mark 3. **Supplementary Fig. 2.** Scatterplots of 6MWT vs. NSAA scores, HUI3 and HUI2 utility values at baseline, with best-fitting lines. Abbreviations: 6MWT, 6-minute walk test; HUI, Health Utilities Index; m: meters; NSAA, North Star Ambulatory Assessment. **Supplementary Fig. 3.** Mean (SD) A HUI utility and B NSAA score by visit and age at baseline. Whiskers represent 95% confidence intervals. Abbreviations: HUI, Health Utility Index; HUI2, Health Utility Index mark 2; HUI3, Health Utility Index mark 3; NSSA, North Star Ambulatory Assessment.

## Data Availability

The data that support the findings of this study are available from Sarepta Therapeutics Inc. but restrictions apply to the availability of these data, which were used under licence for the current study, and so are not publicly available. Data are however available from the authors upon reasonable request and with the permission of Sarepta Therapeutics, Inc.

## Abbreviations

6MWT: 6-Minute walk test
10MWR: 10-Meter walk/run
DMD: Duchenne muscular dystrophy
HRQoL: Health-related quality-of-life
HUI: Health Utilities Index
IQR: Interquartile range
NSAA: North Star Ambulatory Assessment
RFF: Rise from floor
SD: Standard deviation
TFT: Timed function test

## References

[1] Yiu EM, Kornberg AJ (2015). Duchenne muscular dystrophy. J Paediatr Child Health.

[2] Wein N, Alfano L, Flanigan KM (2015). Genetics and emerging treatments for Duchenne and Becker muscular dystrophy. Pediatr Clin North Am.

[3] Mirski KT, Crawford TO (2014). Motor and cognitive delay in Duchenne muscular dystrophy: implication for early diagnosis. J Pediatr.

[4] Connolly AM, Florence JM, Cradock MM, Eagle M, Flanigan KM, McDonald CM, Karachunski PI, Darras BT, Bushby K, Malkus EC (2014). One year outcome of boys with Duchenne muscular dystrophy using the Bayley-III scales of infant and toddler development. Pediatr Neurol.

[5] Connolly AM, Florence JM, Cradock MM, Malkus EC, Schierbecker JR, Siener CA, Wulf CO, Anand P, Golumbek PT, Zaidman CM (2013). Motor and cognitive assessment of infants and young boys with Duchenne Muscular Dystrophy: results from the Muscular Dystrophy Association DMD Clinical Research Network. Neuromuscul Disord.

[6] Szabo SM, Salhany RM, Deighton A, Harwood M, Mah J, Gooch KL (2021). The clinical course of Duchenne muscular dystrophy in the corticosteroid treatment era: a systematic literature review. Orphanet J Rare Dis.

[7] Landfeldt E, Lindgren P, Bell CF, Guglieri M, Straub V, Lochmuller H, Bushby K (2016). Health-related quality of life in patients with Duchenne muscular dystrophy: a multinational, cross-sectional study. Dev Med Child Neurol.

[8] Uttley L, Carlton J, Woods HB, Brazier J (2018). A review of quality of life themes in Duchenne muscular dystrophy for patients and carers. Health Qual Life Outcomes.

[9] Brown V, Merikle E, Johnston K, Audhya I, Gooch K, Lowes L: Qualitative interviews to understand the Duchenne muscular dystrophy (DMD) experience from the caregiver/patient perspective. In Muscular Dystrophy Association (MDA); March 13–16. 2022 10.1186/s41687-023-00669-6 PMC10716079 38085412

[10] Mazzone ES, Messina S, Vasco G, Main M, Eagle M, D'Amico A, Doglio L, Politano L, Cavallaro F, Frosini S (2009). Reliability of the North Star Ambulatory Assessment in a multicentric setting. Neuromuscul Disord.

[11] Scott E, Eagle M, Mayhew A, Freeman J, Main M, Sheehan J, Manzur A, Muntoni F (2012). Development of a functional assessment scale for ambulatory boys with Duchenne muscular dystrophy. Physiother Res Int.

[12] Mayhew A, Cano S, Scott E, Eagle M, Bushby K, Muntoni F (2011). Moving towards meaningful measurement: Rasch analysis of the North Star Ambulatory Assessment in Duchenne muscular dystrophy. Dev Med Child Neurol.

[13] Muntoni F, Domingos J, Manzur AY, Mayhew A, Guglieri M, Sajeev G, Signorovitch J, Ward SJ (2019). Categorising trajectories and individual item changes of the North Star Ambulatory Assessment in patients with Duchenne muscular dystrophy. PLoS One.

[14] Mazzone E, Martinelli D, Berardinelli A, Messina S, D'Amico A, Vasco G, Main M, Doglio L, Politano L, Cavallaro F (2010). North Star Ambulatory Assessment, 6-minute walk test and timed items in ambulant boys with Duchenne muscular dystrophy. Neuromuscul Disord.

[15] Pane M, Mazzone ES, Sivo S, Sormani MP, Messina S, D'Amico A, Carlesi A, Vita G, Fanelli L, Berardinelli A (2014). Long term natural history data in ambulant boys with Duchenne muscular dystrophy: 36-month changes. PLoS One.

[16] Messina S, Vita GL, Sframeli M, Mondello S, Mazzone E, D'Amico A, Berardinelli A, La Rosa M, Bruno C, Distefano MG (2016). Health-related quality of life and functional changes in DMD: A 12-month longitudinal cohort study. Neuromuscul Disord.

[17] Wolowacz SE, Briggs A, Belozeroff V, Clarke P, Doward L, Goeree R, Lloyd A, Norman R (2016). Estimating Health-State Utility for Economic Models in Clinical Studies: An ISPOR Good Research Practices Task Force Report. Value Health.

[18] Gray AM, Clarke PM, Wolstenholme JL, Wordsworth S (2011). Applied Methods of Cost-Effectiveness Analysis in Health Care.

[19] Neumann PJ, Willke RJ, Garrison LP (2018). A Health Economics Approach to US Value Assessment Frameworks-Introduction: An ISPOR Special Task Force Report [1]. Value Health.

[20] Szabo SM, Audhya IF, Malone DC, Feeny D, Gooch KL (2020). Characterizing health state utilities associated with Duchenne muscular dystrophy: a systematic review. Qual Life Res.

[21] Rowen D, Powell P, Mukuria C, Carlton J, Norman R, Brazier J (2021). Deriving a Preference-Based Measure for People With Duchenne Muscular Dystrophy From the DMD-QoL. Value Health.

[22] Landfeldt E, Lindgren P, Bell CF, Schmitt C, Guglieri M, Straub V, Lochmuller H, Bushby K (2014). The burden of Duchenne muscular dystrophy: an international, cross-sectional study. Neurology.

[23] Szabo SM, Audhya IF, Rogula B, Feeny D, Gooch KL (2022). Factors associated with the health-related quality of life among people with Duchenne muscular dystrophy: a study using the Health Utilities Index (HUI). Health Qual Life Outcomes.

[24] Lans C, Cider A, Nylander E, Brudin L (2022). The relationship between six-minute walked distance and health-related quality of life in patients with chronic heart failure. Scand Cardiovasc J.

[25] Modaresi M, Roshanzamir Z, Shirzadi R (2022). The Correlation of Health-Related Quality of Life with Cystic Fibrosis Severity Markers in Chest CT Scan and 6-Minute Walk Test: A Cross-Sectional Study. Indian J Pediatr.

[26] O'Dowd DN, Bostock EL, Smith D, Morse CI, Orme P, Payton CJ (2021). Psychological parameters impact health-related quality of life in mental and physical domains in adults with muscular dystrophy. Neuromuscul Disord.

[27] Yagi K, Asakura T, Namkoong H, Suzuki S, Asami T, Okamori S, Kusumoto T, Funatsu Y, Kamata H, Nishimura T (2018). Association between six-minute walk test parameters and the health-related quality of life in patients with pulmonary Mycobacterium avium complex disease. BMC Pulm Med.

[28] A Clinical Study to Assess the Efficacy and Safety of GSK2402968 in Subjects With Duchenne Muscular Dystrophy (DMD114044) [https://clinicaltrials.gov/ct2/show/NCT01254019].

[29] Horsman J, Furlong W, Feeny D, Torrance G (2003). The Health Utilities Index (HUI®): concepts, measurement properties and applications. Health Qual Life Outcomes.

[30] Furlong WJ, Feeny DH, Torrance GW: Health Utilities Index (HUI) procedures manual: Algorithm for determining HUI mark 2 (HUI2)/mark 3 (HUI3) health status classification levels, health states, single-attribute level utility scores and overall health-related quality of life utility scores from 40-item Interviewer-administered health states questionnaires (also referred to as HUI23.40Q questionnaires). Dundas, Ontario, Canada: Health Utilities Inc.; 2012

[31] Mazzone E, Vasco G, Sormani MP, Torrente Y, Berardinelli A, Messina S, D'Amico A, Doglio L, Politano L, Cavallaro F (2011). Functional changes in Duchenne muscular dystrophy: a 12-month longitudinal cohort study. Neurology.

[32] Haberkamp M, Moseley J, Athanasiou D, de Andres-Trelles F, Elferink A, Rosa MM, Magrelli A (2019). European regulators' views on a wearable-derived performance measurement of ambulation for Duchenne muscular dystrophy regulatory trials. Neuromuscul Disord.

[33] Mayhew AG, Cano SJ, Scott E, Eagle M, Bushby K, Manzur A, Muntoni F (2013). North Star Clinical Network for Neuromuscular D: Detecting meaningful change using the North Star Ambulatory Assessment in Duchenne muscular dystrophy. Dev Med Child Neurol.

[34] Sciurba FC, Slivka WA (1998). Six-Minute Walk Testing. Semin Respir Crit Care Med.

[35] Szabo SM, Audhya IF, Bever A, Feeny D, Malone DC, Neumann PJ, Mickle AM, Iannaccone ST, Gooch KL: The impact of key health state transitions on health-related quality of life in Duchenne muscular dystrophy: A qualitative study. In ISPOR. 2022

[36] Powell PA, Carlton J, Rowen D, Chandler F, Guglieri M, Brazier JE (2021). Development of a New Quality of Life Measure for Duchenne Muscular Dystrophy Using Mixed Methods: The DMD-QoL. Neurology.

[37] Wang RT, Barthelemy F, Martin AS, Douine ED, Eskin A, Lucas A, Lavigne J, Peay H, Khanlou N, Sweeney L (2018). DMD genotype correlations from the Duchenne Registry: Endogenous exon skipping is a factor in prolonged ambulation for individuals with a defined mutation subtype. Hum Mutat.

