# Supplementary material for: Exploring the relationship between North Star Ambulatory Assessment and Health Utilities Index scores in Duchenne muscular dystrophy
Source: Health Qual Life Outcomes. 2023 Jul 19;21:76. doi: 10.1186/s12955-023-02160-8 (PMC10355009; doi:10.1186/s12955-023-02160-8)

**SUPPLEMENTARY MATERIALS**

Supplementary Table

Measure scoring

| **Timed 10MWR** | |
| --- | --- |
| **Grade** | **Description** |
| 1 | Unable to walk independently |
| 2 | Unable to walk independently but can walk with KAFOs or support from person |
| 3 | Highly adapted wide based lordotic gait. Cannot increase walking speed |
| 4 | Moderately adapted gait. Can pick up speed but cannot run |
| 5 | Able to pick up speed, but runs with double stance phase |
| 6 | Runs and gets off both feet off the ground (with no double stance phase) |
| **RFF** | |
| **Grade** | **Description** |
| 1 | Unable to stand from supine, even with use of a chair |
| 2 | Assisted Gowers: requires furniture for help in arising from supine to upright |
| 3 | Full Gowers: rolls over, stands up with both hands "climbing up” legs to upright |
| 4 | Half Gowers: rolls over, stands up with one hand support on leg |
| 5 | Rolls to side and/or stands up with hands on floor to rise, does not touch legs |
| 6 | Stands up without rolling over |
| **NSAA scores** | |
| NSAA scores range from 0 to 34 and are calculated as a sum of 17 activity grades, each with possible values 0 (unable to achieve independently), 1 (modified method but achieves goal independent of physical assistance from another), or 2 (normal – no obvious modification of activity) | |

Abbreviations: 10MWR, 10-meter walk/run; KAFO, knee-ankle-foot orthoses; NSAA, North Star Ambulatory Assessment; RFF, rise from floor.

Supplementary Fig. 1. HUI scoring. *HUI2 level codes for Sensation, Mobility, and Cognition attributes can be derived directly from HUI3 attribute level codes. Abbreviations: HUI, Health Utility Index; HUI2, Health Utility Index mark 2; HUI3, Health Utility Index mark 3.


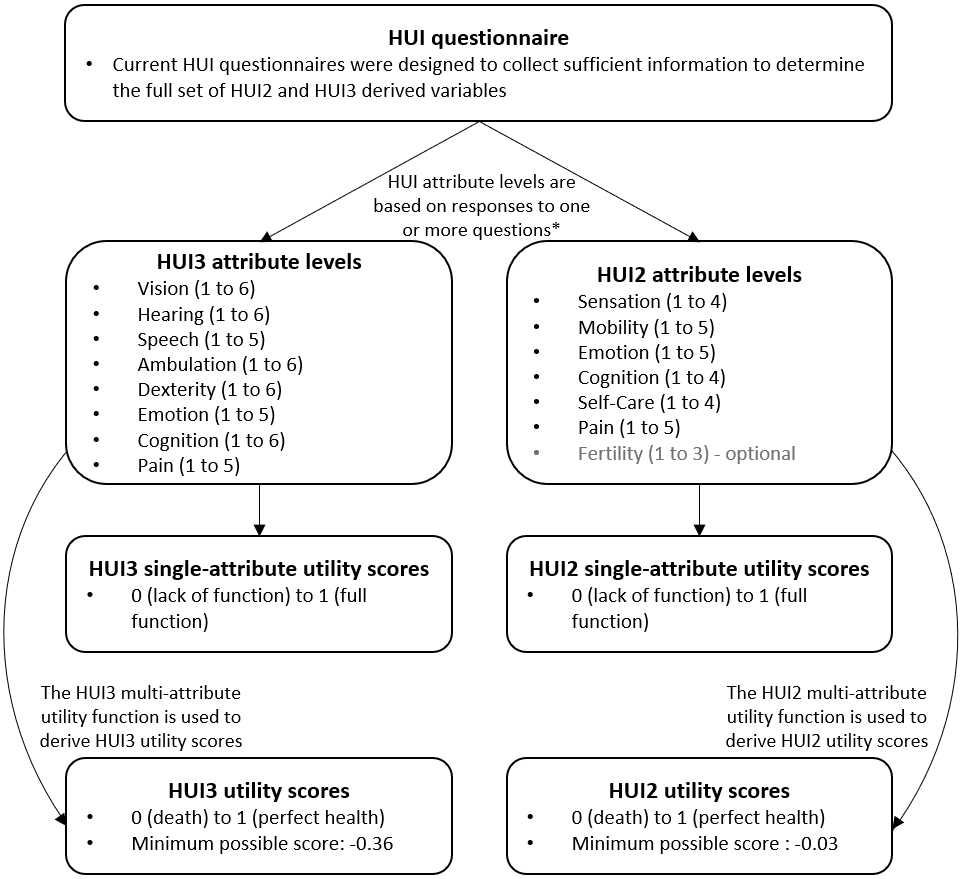


Supplementary Fig. 2. Scatterplots of 6MWT vs. NSAA scores, HUI3 and HUI2 utility values at baseline, with best-fitting lines. Abbreviations: 6MWT, 6-minute walk test; HUI, Health Utilities Index; m: meters; NSAA, North Star Ambulatory Assessment.


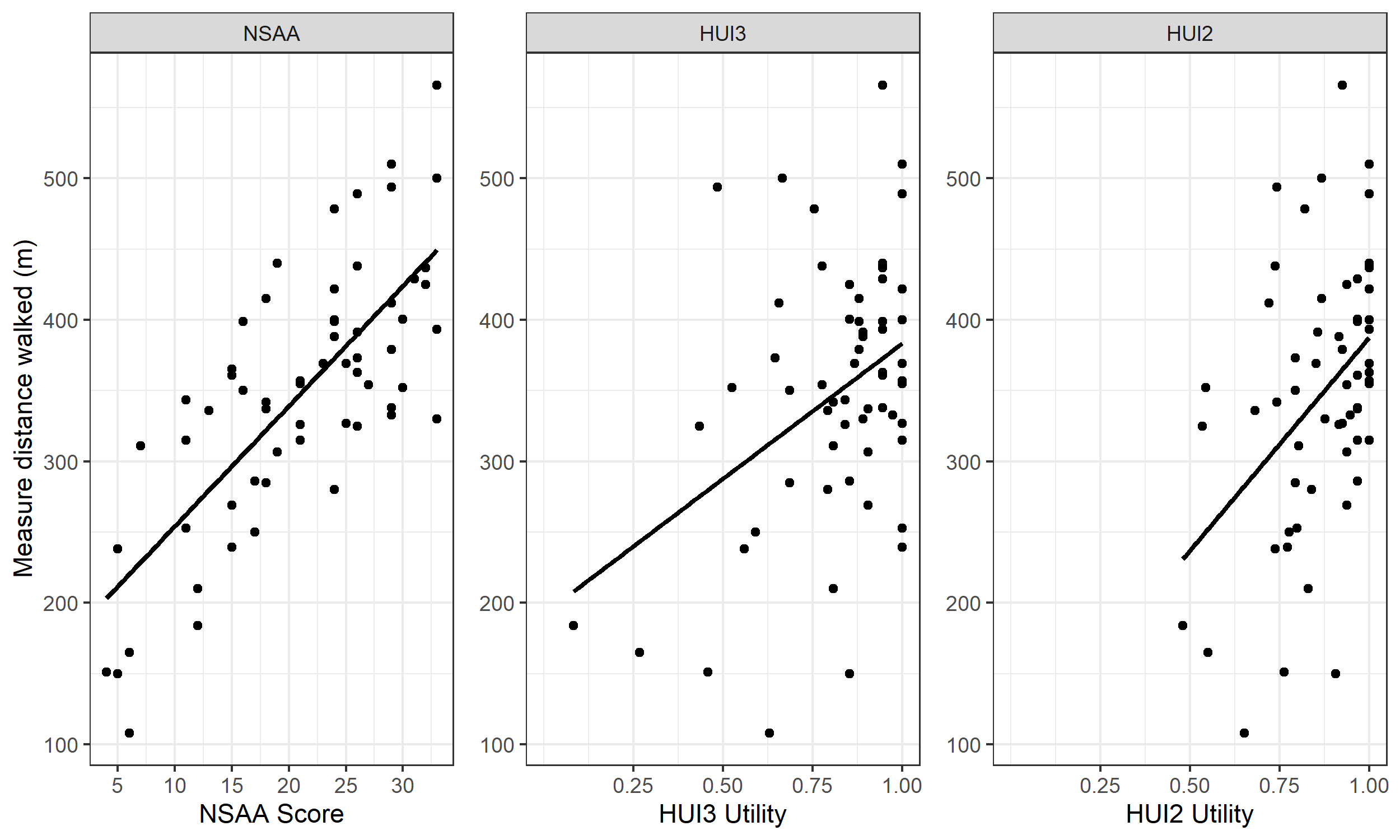


Supplementary Fig. 3. Mean (SD) **A** HUI utility and **B** NSAA score by visit and age at baseline. Whiskers represent 95% confidence intervals. Abbreviations: HUI, Health Utility Index; HUI2, Health Utility Index mark 2; HUI3, Health Utility Index mark 3; NSSA, North Star Ambulatory Assessment.


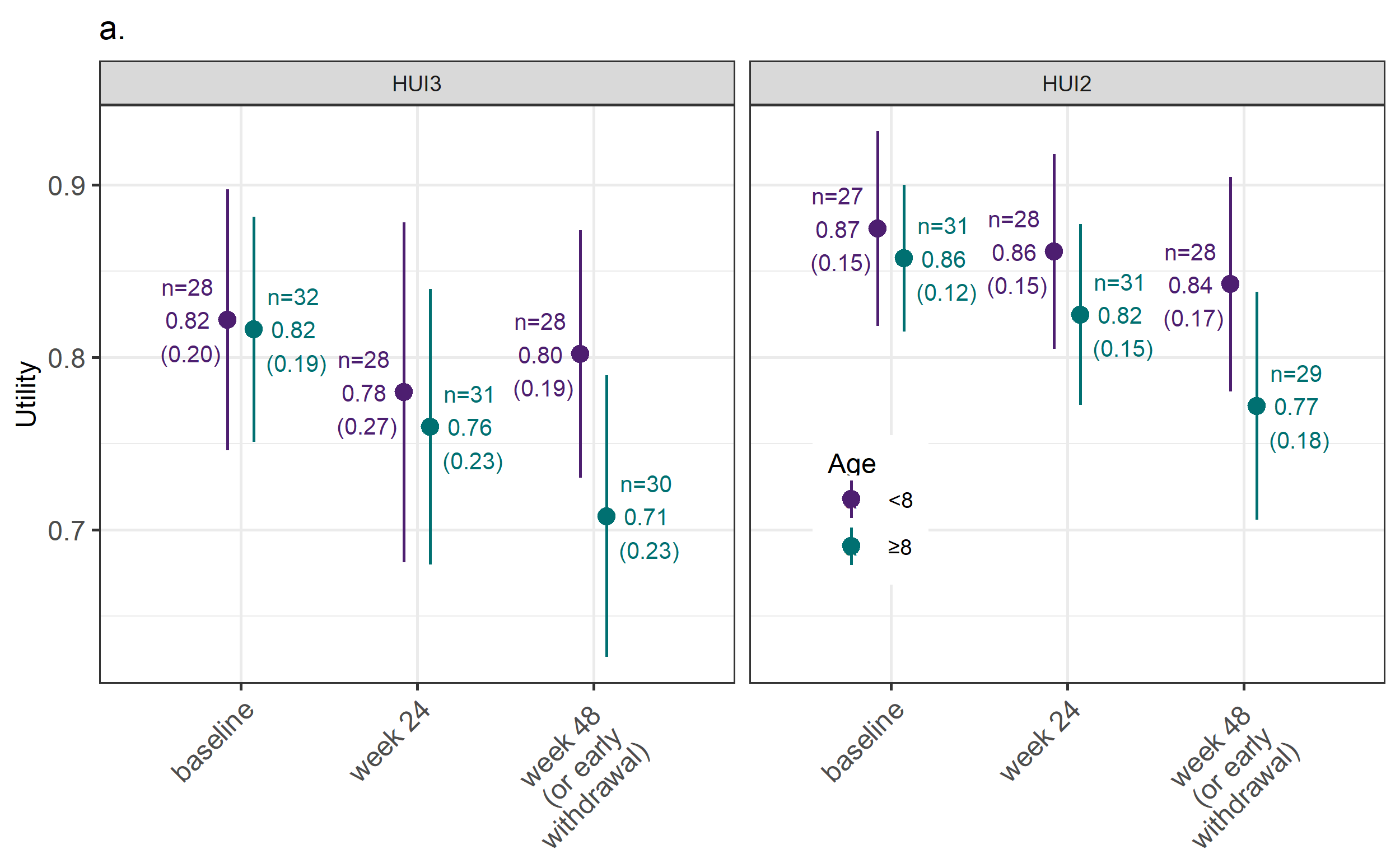

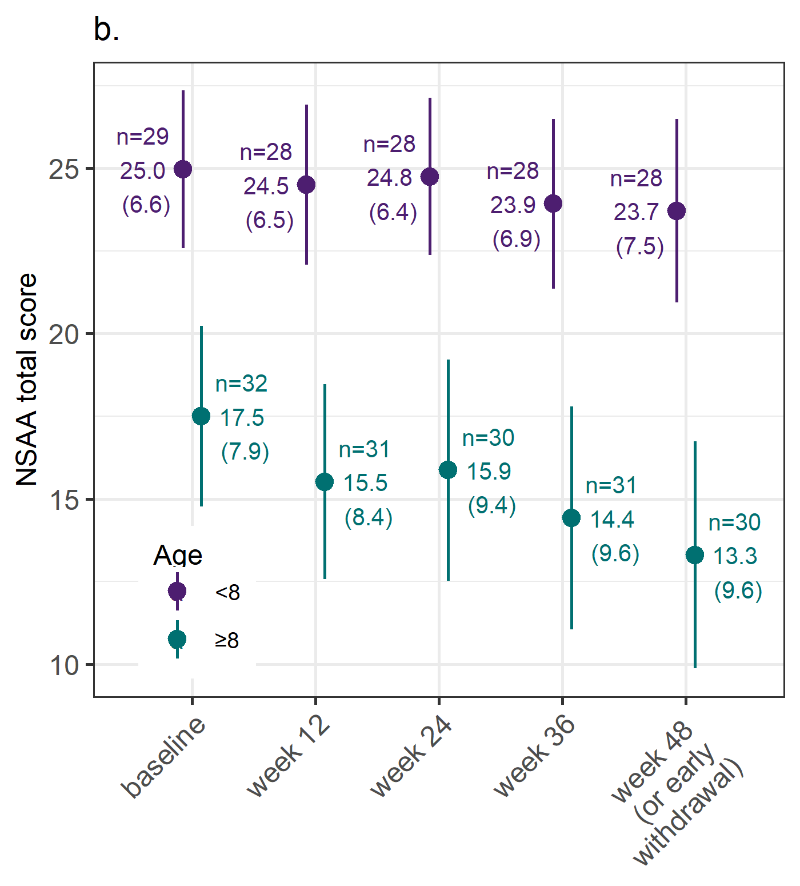

Supplement: Supplementary file 1 — Additional file 1: Supplementary Table. Measure scoring. Supplementary Fig. 1. HUI scoring. *HUI2 level codes for Sensation, Mobility, and Cognition attributes can be derived directly from HUI3 attribute level codes. Abbreviations: HUI, Health Utility Index; HUI2, Health Utility Index mark 2; HUI3, Health Utility Index mark 3. Supplementary Fig. 2. Scatterplots of 6MWT vs. NSAA scores, HUI3 and HUI2 utility values at baseline, with best-fitting lines. Abbreviations: 6MWT, 6-minute walk test; HUI, Health Utilities Index; m: meters; NSAA, North Star Ambulatory Assessment. Supplementary Fig. 3. Mean (SD) A HUI utility and B NSAA score by visit and age at baseline. Whiskers represent 95% confidence intervals. Abbreviations: HUI, Health Utility Index; HUI2, Health Utility Index mark 2; HUI3, Health Utility Index mark 3; NSSA, North Star Ambulatory Assessment. [file 12955_2023_2160_MOESM1_ESM.docx]
